# Sphere-plane methodology to evaluate the wear of titanium of dental implants: a research proposal

**DOI:** 10.1186/s13104-018-3635-8

**Published:** 2018-07-31

**Authors:** Teresa Almeida Mendes, João Caramês, Luís Pires Lopes, Amílcar Lopes Ramalho

**Affiliations:** 1 Faculdade de Medicina Dentária, Universidade de Lisboa, Cidade Universitária, 1649-003 Lisbon, Portugal; 2 CEMMPRE, Department of Mechanical Engineering, University of Coimbra, Rua Luís Reis Santos, 3030-788 Coimbra, Portugal

**Keywords:** Dental implants, Wear, Radial fretting, Abutment, Titanium, Zirconia

## Abstract

**Objective:**

Titanium is the most commonly used material to manufacture dental implants and abutments. Recently, zirconia abutments have been manufactured with better aesthetic properties. However, zirconia abutments are harder than titanium implants; therefore, they could wear the implant surface. Therefore, this article aims to describe a sphere-plane system that can be used to assess the wear that different abutment materials cause in the titanium of dental implants when submitted to cyclic loading. This method can be used to simulate the oral cavity, where the abutment (sphere) applies loads onto the implant (titanium plane). The spheres were made of different materials (titanium and zirconia), and the specimens were loaded for 4,000,000 cycles. The scar size and area on titanium planes were measured with stereoscopic images and analysed through profilometry.

**Results:**

The wear of titanium planes was similar when tested against zirconia or titanium spheres. The sphere-plane system is a method that can be used to evaluate and quantify the wear of the titanium of dental implants, and compared with methods that use real implants, this system is simpler and less expensive. This method could facilitate further research to evaluate the wear of titanium against different materials and under different testing conditions.

**Electronic supplementary material:**

The online version of this article (10.1186/s13104-018-3635-8) contains supplementary material, which is available to authorized users.

## Introduction

The most commonly used material to manufacture implants and abutments is titanium [1]. Titanium is a biocompatible material [2] with a low risk of corrosion [3]. However, titanium abutments can change the appearance of soft tissues, thereby compromising aesthetics [4, 5].

To overcome this limitation, zirconia abutments were manufactured (polycrystalline tetragonal zirconium oxide, stabilized with 3-yttrium) [6]. Since the introduction of zirconia abutments, the interest in them has greatly increased due to their aesthetic properties and high mechanical strength [2]. However, according to several authors, zirconia abutments could be more prone to fracture [7] and have less marginal accuracy [8] than titanium abutments. As zirconia is 10 times harder than titanium [2], it could cause wear on the implant surface over time [9, 10]. This phenomenon is called fretting wear and can compromise the long-term stability of the implant-abutment connection [10].

Radial fretting wear is caused by the relative oscillatory movement of small amplitudes, which may occur between two surfaces that remain in contact [11] and is primarily induced by varying the normal load [12]. In radial fretting, the cyclic load is applied to a Hertzian contact, resulting in a cyclic increase and decrease of the contact area. The morphology of the contact surface displays wear in an annular region, which corresponds to a region of slip between the two materials in contact [13].

This technique can be used to simulate the implant abutment’s contact with a sphere-plane system, where the sphere simulates the abutment and the plane simulates the implant. As in the implant-abutment surface, in the sphere-plane system, the surfaces remain in contact, and the relative oscillatory movement has a small amplitude. Micromotion at the implant-abutment interface ranged from 1.52 to 94.00 μm at the external contact [14]; in the internal contact, it varied between 0.20 and 5.67 μm [15] with different types of implant-abutment connections (external and internal).

The geometry of a real implant-abutment connection is complex, making it difficult to obtain data from the implant to measure the wear [9]. To the best of our knowledge, only one study [9] described data from the wear in a dental implant at the implant-abutment connection using a very complex and expensive methodology. The objective of this article is to describe a new method that uses a sphere-plane system with simple geometry to measure the wear and fatigue phenomena that different abutment materials can cause in the titanium of dental implants when submitted to cyclic loading.

## Main text

### Methods

This in vitro study used a sphere-plane contact. The spheres applied cyclic loads on titanium planes in a mechanically actuated contact fatigue and radial fretting test machine. This setup was used with the intention of simulating oral cavity use, where the abutment (sphere) transfers the masticatory forces to the dental implant (plane).

#### Materials

The planes were made of commercially pure titanium grade 4 (ASTM F67) [16]. Titanium grade 4 has the highest strength of all of the unalloyed ASTM pure titanium grades [17] and is considered the material of choice for intra-osseous use in the medical field [18]. Titanium grade 4 is less toxic than titanium alloys and has a low allergenic potential and an excellent corrosion resistance [18]. The minimization of ion release contributes to the biocompatibility of this material [2].

Two different sphere materials were tested to simulate implant abutments, forming two different groups: titanium grade 5 (control group) and zirconia [6] (experimental group). The material most commonly used to manufacture restorative abutments is titanium grade 5 (Ti-6Al-4V), since this alloy has higher hardness and fatigue resistance in comparison with pure titanium [17].

Titanium planes were polished following the regimen recommended for metallographic polishing [19, 20].

#### Testing conditions and cyclic load

The testing machine was a tribological system with mechanically actuated contact fatigue and radial fretting built at the Department of Mechanical Engineering, University of Coimbra. A relative displacement was generated between the spherical specimen placed on the top (sphere vertical support) and the flat specimen (horizontal) in accordance with the flexible vibration of the electric motor (Additional file 1).

For each group, tests were performed under two different simulation environments: air and artificial saliva [21]. The test performed in air was used to control the influence of artificial saliva (SAGF medium), which enables the in vitro reproduction and the study of many physicochemical phenomena that can develop in the mouth. It has a complete composition closer to human saliva than other means of simpler artificial saliva [21].

The artificial saliva was applied twice a day (12 h of interval) to the interface of the plane-sphere with a PVC tube with a diameter of 4.2 cm. The SAGF pH was checked every day (Inolab WTW) to determine if they were stable.

The specimens were submitted to a 20 N load (peak to peak) with a 15 Hz frequency for 4,000,000 cycles with a sphere measuring 10 mm in diameter. Table 1 presents the four samples that were tested and the testing conditions that were met, with one specimen being tested in each testing condition.

**Table 1:** Samples tested

Plane (substrate)	Sphere (abrader)	Testing conditions
Commercially pure titanium Grade 4	Control group Titanium Gr5	Room atmosphere
		Artificial Saliva
	Experimental group Zirconia	Room atmosphere
		Artificial Saliva

#### Area loss and scar measurement

The geometry of the planes with damaged contact areas was measured with a laser profilometry microtopographer (Mahr, Germany). The data obtained from the microtopographer was analysed with *Gwyddion (Czech Metrology Institute),* a modular program for data visualization and analysis, and the two-dimensional (2D) profiles of the scars were extracted. The concavities on the edges of the scars (wear zones) were further analysed with Microsoft *Excel w*ith the tool Curve integration Macro to determine the removed area by calculating the area on the concave zone of the scar.

Additionally, images of the scars on planes were obtained with the stereoscopic microscope (Nikon Stereophoto SMZ-10) and analysed with *ImageJ software (National Institutes for Health)* to measure the outer diameter. Five measures were obtained for each specimen.

#### Scanning electron microscope (SEM) images

SEM images of the scars on planes were obtained with low and high magnification (Philips XL series 30).

#### Hertzian diameter

The contact diameter between the plane and sphere can be calculated as the Hertzian diameter. The following formula is used for the calculation:

$$\alpha = \left(\frac{3P{R_{eq}}}{4E^{*}}\right)^{\frac{1}{3}}$$

*Formula 1—Hertzian diameter* P represents the normal force; R_eq_ is the reduced radius, and E is the reduced Young’s modulus of the materials.

### Results

#### Stereoscope images of titanium plane scars

**Fig. 1:**
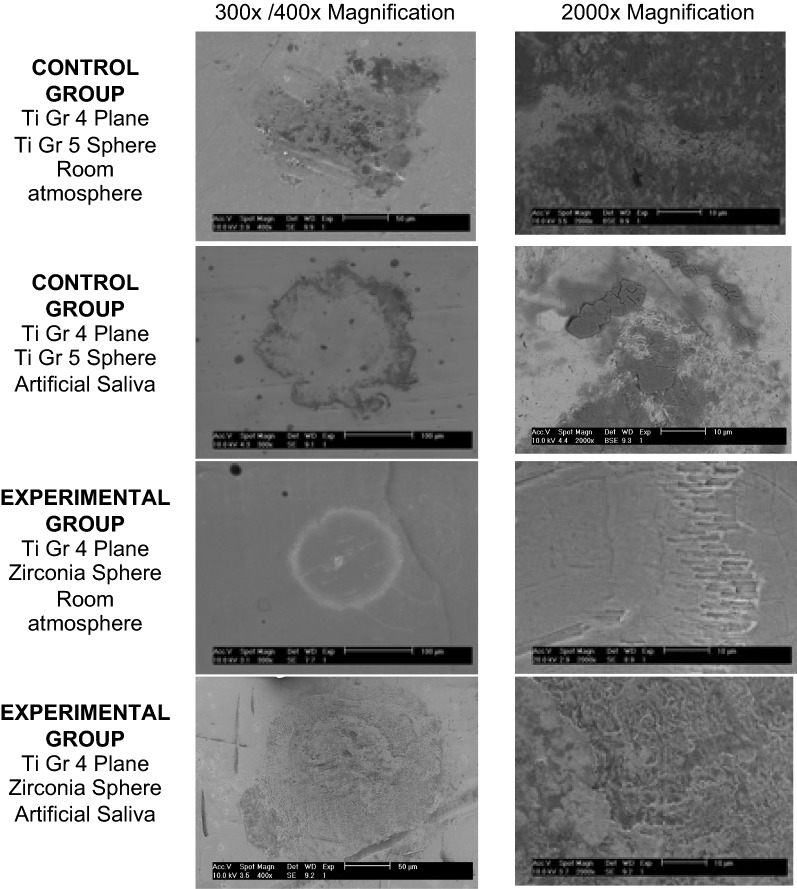
SEM images of titanium grade 4 plane scars

Representative SEM images of titanium plane scars are depicted in Fig. 1. The configuration of the fretting wear scars left on the planes is similar to a ring in a top view. With the zirconia sphere at room temperature, the ring scar is the most perfect. On the test performed with artificial saliva in SEM images at high magnification, we can see that the ring surface is highly irregular and dense.

#### Area loss on titanium planes

The material area loss was low on the control and experimental groups, with the area of loss between 0.10 and 0.03 µm^2^ on the test performed with titanium–titanium contact at room temperature and in contact with artificial saliva, respectively. Regardless of the material used in the sphere, the amount of area loss was lower in the presence of artificial saliva.

#### Diameter of the scars on titanium planes

**Fig. 2:**
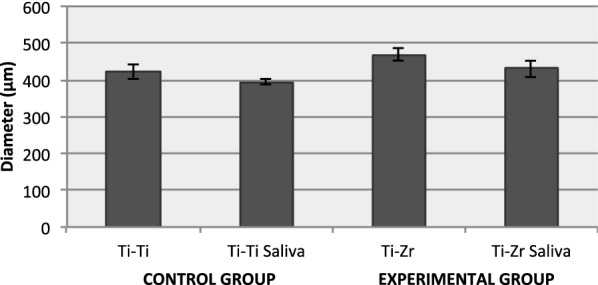
Results of average and standard deviation of the outer diameter (out) of titanium grade 4 plane scars

The graphs in Fig. 2 represents the outer diameter of the scars measured on titanium planes. The outer diameter was similar in control and experimental groups. At room temperature, the outer diameter was slightly higher than that of the tests performed under artificial saliva.

#### Hertzian diameter

The contact diameter between the plane and sphere varied between 0.13 and 0.23 mm.

### Discussion

The primary conclusion of this article is that the proposed technique is an alternative for the evaluation of wear on dental implants. It was possible to observe and to measure the wear, which was different according to the antagonist surfaces (zirconia and titanium) and the presence and absence of saliva, which means that this method enables the evaluation of different variables (material, presence of saliva).

It can be observed that the tests performed with artificial saliva had smaller scars. The reduction in wear can be explained by the fact that the presence of saliva creates a corrosive environment, which leads to oxide formation that increases the friction coefficient, thus decreasing wear [22]. The scars produced on titanium planes had small differences in diameter and area of loss, both with zirconia (experimental group) and titanium (control group) spheres.

When assessing the obtained data in the 2D profile, it was possible to observe that wear occurs only on the ring corresponding to the slip zone and the inner part of the ring remains almost intact, corresponding to a stick zone. The contact centre is in compression all the time (sticking) and the micro-slip occurs at the contact edge. This displacement field confirms the existence of radial fretting that always runs in partial slip contact [13].

According to the results of this test, the expected area of loss on titanium planes that simulate dental implants will be under 0.1 µm^2^. These planes require accurate image acquisition and data analysis tools. The internal geometry of a dental implant is complex due to the indexations that are intended to create anti-rotational resistance and serve as a reference for the positioning of the prosthetic abutment. The implant-abutment connections can be divided into two major groups: external and internal connections. The internal connections were introduced more recently. The connections are characterized by the presence of the connection mechanism inside the implant body [23]. Internal connections have a superior contact area and lower micromovements between implant and abutment [24]. These movements may cause wear of dental implants [10], which may be lower on internal connections. Further studies are needed to increase the knowledge of these phenomena.

The geometry of internal connections is challenging to observe. In electron microscopy, the interior of the implants appears as a deep, unfocused dark cavity. Even in optical 3D surface metrology (Alicona, Austria) coupled with light devices, the images obtained have many imperfections due to the light reflection from the metal of the implant.

Recent studies measured the implant abutment system by longitudinal cross-sections after embedding the system in epoxy resin. A mechanical means of sectioning and polishing to enable direct visualization of the implant-abutment interface can produce significant deformation on the surface of the specimen, smearing the materials and polishing residue into the spaces between the components [25].

The force selected for this test was 20 N to prevent the plastic deformation that can occur between the contact surfaces when submitted to stronger forces, thereby removing the radial fretting [11]. The contact diameter between the plane and sphere (Hertzian diameter) was 0.13 to 0.23 mm. An implant with a regular platform is 4.1 mm in diameter; therefore, the contact between the implant and the abutment has that diameter, which is greater than the diameter of the sphere-plane contact. In the mouth, there are forces superior to 20 N [26], but the diameter of contact between the surfaces is greater.

### Conclusions

The sphere-plane system enables the simulation of the dental implant-abutment contact. This system is an alternative method for the evaluation of wear on dental implants that is simpler and less expensive than the methods that use real implants. The implant is represented by the plane with simple geometry, enabling accurate measurement of the wear with profilometry techniques and SEM images. With this method, it is possible to evaluate the wear with different abutment materials and different testing environments, including artificial saliva.

## Limitations

Further tests should be improved with a greater sphere diameter to create larger scars on titanium planes to be easily measured with 3D profilometry and measure the volume of loss.

As an in vitro study, these conditions were a simulation of the oral environment. Further microscopy techniques should be developed by microscope manufacturers to improve the measurements of the real geometry with different implant connections.

## Additional file

**Additional file 1: Figure S1.** Schematic representation of the testing machine and correspondence between the sphere-plane system and implant-abutment system. This figure contains a schematic representation of the testing machine used on the article. It also shows the correspondence between the sphere and a prosthetic abutment, and between the plane and a dental implant.
