# Supplementary figures and images for: Sphere-plane methodology to evaluate the wear of titanium of dental implants: a research proposal
Source: BMC Res Notes. 2018 Jul 31;11:529. doi: 10.1186/s13104-018-3635-8 (PMC6069542; doi:10.1186/s13104-018-3635-8)

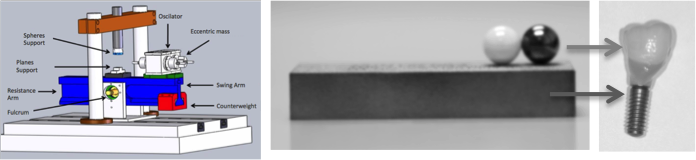

Supplement: Supplementary file 1 — Additional file 1: Figure S1. Schematic representation of the testing machine and correspondence between the sphere-plane system and implant-abutment system. This figure contains a schematic representation of the testing machine used on the article. It also shows the correspondence between the sphere and a prosthetic abutment, and between the plane and a dental implant. [file 13104_2018_3635_MOESM1_ESM.png]
